# *Streptococcus pyogenes* Endocarditis Associated With Varicella—Case Report and Review of the Literature

**DOI:** 10.3389/fped.2019.00500

**Published:** 2019-12-04

**Authors:** Patrizia Savoia, Ulrich Heininger, Michael Buettcher

**Affiliations:** ^1^Paediatric Intensive Care and Neonatology, Lucerne Children's Hospital, Cantonal Hospital Lucerne, Lucerne, Switzerland; ^2^Department of Paediatric Infectious Diseases and Vaccinology, University of Basel Children's Hospital, Basel, Switzerland; ^3^Paediatric Infectious Diseases, Lucerne Children's Hospital, Cantonal Hospital Lucerne, Lucerne, Switzerland

**Keywords:** varicella, *Streptococcus pyogenes*, necrotizing fasciitis, infective endocarditis, mitral valve, vaccination

## Abstract

Infection with varicella zoster virus (VZV) is usually a benign and self-limiting disease. Serious complications by bacterial pathogens do occur, such as necrotising fasciitis (NF). One of the most important is *Streptococcus pyogenes* (or Group A Streptococcus, GAS), which colonizes epithelial surfaces, primarily of the throat and skin. In rare instances, varicella may also be associated with *S. pyogenes* endocarditis. Review of the literature reveals only 18 children with infective endocarditis (IE) caused by GAS since 1942. VZV as antecedent illness was found in five (28%). Fourteen (78%) had no pre-existing cardiac abnormalities. Death occurred in three (17%) children. Infective endocarditis with acute deterioration secondary to rupture of mitral valve chordae tendineae necessitating cardiac surgery has not been reported in the literature yet. We present this rare and instructive pediatric case and the results of a literature search on *S. pyogenes* endocarditis in children.

## Background

Varicella-zoster virus (VZV) is the causative agent of varicella, a common infectious disease in children which is usually benign and self-limiting. However, various serious and potentially lethal complications (e.g., bacterial skin and soft-tissue infections, pneumonia, encephalitis, cerebral vasculopathy, myocarditis) do occur occasionally and may lead to hospitalization or death (1). Bacterial superinfection of the skin is reported in up to 45% of hospitalized varicella cases (2, 3). In Europe annual incidence rates for varicella vary from 300 to 1291 per 100,000 population (4). In Switzerland the hospitalization rate for varicella in children and adolescents has been estimated at 1.8 per 100,000 per year in the absence of widespread vaccination (5). In other European countries these incident rates range from 0.1 to 68 per 100,000 per year with children <5 years contributing the highest rates (6). In the USA hospitalization rates dropped from 31 to 15 per 100,000 per year after introduction of universal varicella vaccination (4).

One of the most important bacterial agents in the scope of varicella associated bacterial complications is *Streptococcus pyogenes* (or Group A Streptococcus, GAS) infecting skin, soft tissue, lung, bones and joints (7).

VZV infection increases the risk of invasive GAS infection by 58-fold and is furthermore a significant risk factor for necrotizing fasciitis (NF) (8). In NF penetration of antibiotics into affected tissues is poor, likely due to the acidic and hypoxic environment, facilitating further bacteraemic spread of GAS to other organs (9). GAS has rarely been described as a cause of infective endocarditis (IE). Most IE cases published in the pediatric age group were caused by *S. aureus* in patients with underlying cardiac morbidities (10, 11).

## Case

**Figure 1:**
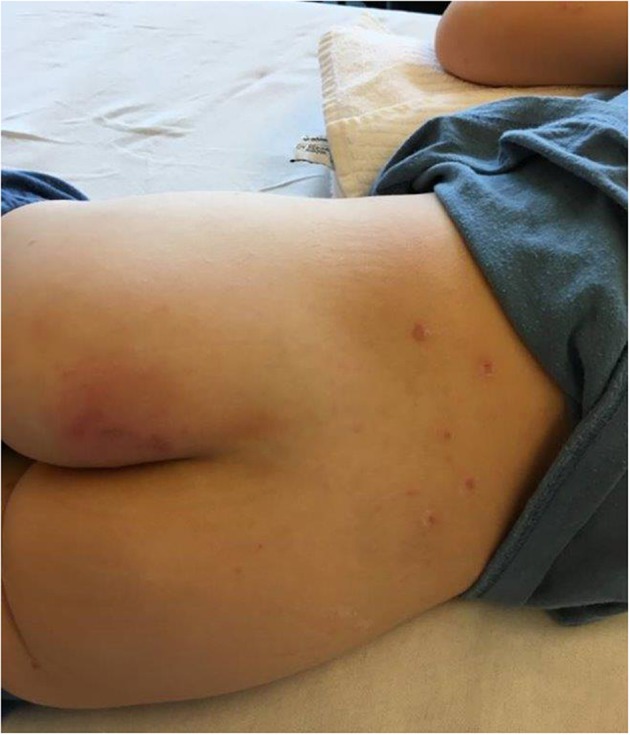
Crusted VZV lesions (sacrum) and reddish-blue painful lesion on left buttock.

A 5-year-old previously healthy, VZV unvaccinated boy presented to our emergency department with typical varicella skin lesions which had developed 2 days prior. He had a history of fever and poor oral intake. Furthermore, he complained of pain around the left thigh and was reluctant to bear weight. The child was in a mildly reduced general condition with normal heart rate, respiratory rate and blood pressure for age. He was febrile with a temperature of 39.6°C. The cardiopulmonary examination was unremarkable. Next to multiple crusted skin lesions there was a tender and discolored area (3–5 cm) on the left buttock (Figure 1). The boy refused to sit or lie on his back.

**Figure 2:**
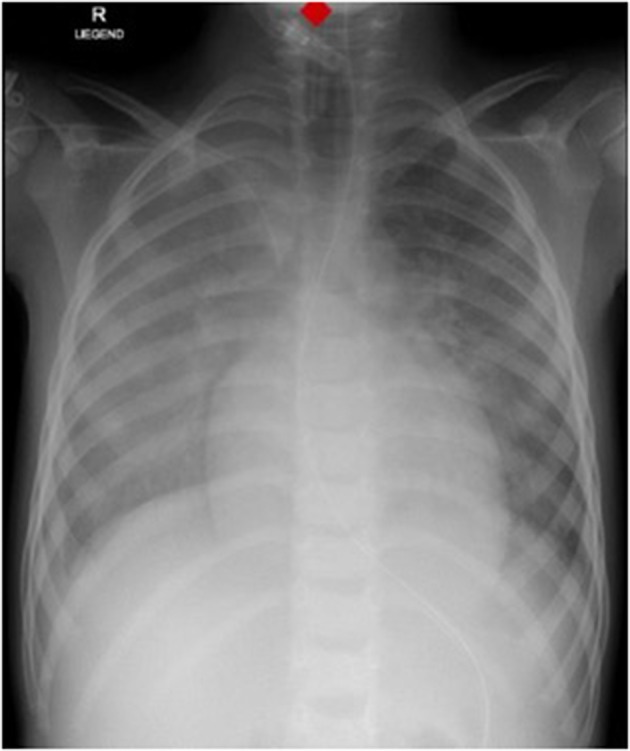
Chest X-Ray showing pulmonary infiltrates and cardiomegaly.

**Figure 3:**
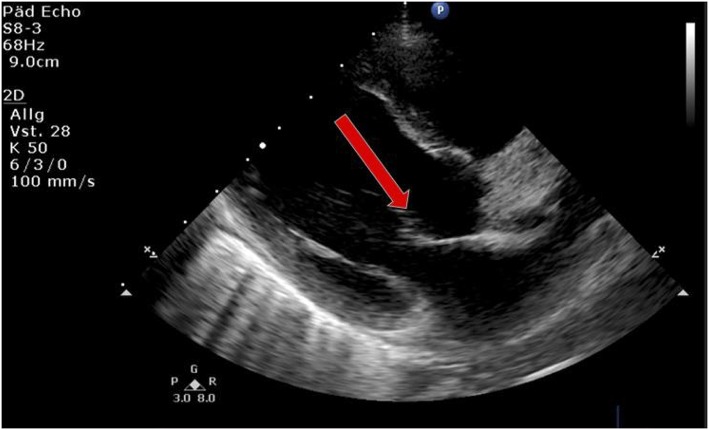
Echocardiography showing prolapse of mitral valve (arrow).

Laboratory work-up showed a white blood cell count (WBC) of 7.2 G/L, platelets of 131 G/L and a CRP of 195 mg/L. Blood cultures were drawn and intravenous Cefuroxime and Clindamycin were started for suspected bacterial soft tissue infection. Growth of GAS from the blood culture was reported with a time to positivity of 2.6 h by the microbiology laboratory. Ultrasound showed signs of soft tissue inflammation around the painful area at the buttock. On the second day of hospitalization the patient had progressively worsening pain of the left thigh. A CT scan revealed inflammation and swelling of the gluteal muscle. Urgent surgical debridement was performed and intraoperatively necrotising fasciitis was confirmed. Tissue swabs grew GAS. Although antimicrobial treatment was started promptly, GAS was still detected in the tissue samples 48 h after initiating betalactam and lincosamide antibiotics at the first debridement. Further blood cultures were not taken at this time. As there was little improvement during the following days, an MRI was performed showing multiple abscesses in the gluteal muscle but no osseous involvement. Overall the child needed two further debridements on days 3 and 4 of hospitalization with application of a vacuum assisted closure (V.A.C.) therapy. On day 5 of hospitalization the patient presented with respiratory distress and required supplementary oxygen. He was transferred to the Pediatric Intensive Care Unit (PICU). On clinical examination a new systolic murmur was heard. Echocardiography revealed mitral valve prolapse with regurgitation. Assuming an endovascular infectious complication, a further set of blood cultures was drawn (which remained sterile) and antibiotic treatment was changed empirically to Gentamycin and Ceftriaxone. Two days later the boy's general condition deteriorated further and a second echocardiography revealed progressive prolapse of the mitral valve, assuming rupture of the chordae tendineae (Figure 2). X-ray of the chest revealed pulmonary infiltrations due to mitral regurgitation (Figure 3). The child was intubated and transferred to a tertiary pediatric cardiac surgery center where the mitral valve was reconstructed the next day and neo-chordae were implanted. Endocarditis was confirmed intraoperatively (small proliferative inflammatory changes of the endocardial tissue) and antibiotic treatment was adjusted to intravenous amoxicillin and continued for 4 weeks. At the day of transfer to the cardiac surgery tertiary center, CRP was 32 mg/l, WBC 15.9 G/L and the child was afebrile. The last documented laboratory findings after 4 weeks of antibiotic treatment showed a CRP <4 and WBC 3.97 G/L and a blood sedimentation rate of 28 mm/h. The wound on the buttock was successfully closed 2 weeks after placement of the V.A.C. Four and a half weeks after primary admission the patient was discharged home in good clinical condition. Cardiology follow-up 1 month later revealed good biventricular function and only mild mitral regurgitation. Screening investigations for an underlying immunodeficiency (quantitative and qualitative humoral and cellular testing and HIV screen) were unremarkable. *S. pyogenes* M serotyping was not done by our laboratory.

## Literature Review

We used MEDLINE to search the English- German and French -language literature from 1942 to 2019 for articles using the following search terms: Infective endocarditis, endocarditis, bacteraemia, varicella, and group A streptococcus or *Streptococcus pyogenes* or GAS. We excluded adults. We focused on antecedent illnesses and cardiac outcome.

## Discussion

The clinical spectrum of varicella ranges from self-limiting disease to potentially life-threatening complications, most of which affect preschool-aged children in the absence of a childhood vaccination program (1, 6). Bacterial skin and soft-tissue infections contribute a large proportion of complications, with GAS being the leading pathogen (5, 7). In adult patients, risk factors for invasive GAS diseases include immunodeficiency states, intravenous drug abuse and comorbidities of different organs. In children however infection with VZV is an important risk factor (12). Skin lesions caused by VZV disrupt its integrity, potentially providing portals for bacterial entry into the bloodstream. On the other hand, VZV infection causes a temporary acquired immunodeficiency with impairment of various immunologic functions of cellular immunity (13, 14). *S. pyogenes* on its own has a large repertoire of virulence factors which can be expressed depending on bacterial transcriptome modifications. The M protein gene (*emm*) encodes the cell surface M virulence protein, responsible for at least 100 *S. pyogenes* M serotypes. Virulence factors can facilitate optimal adherence and colonization to cells and tissues, resistance to the innate immune system (particularly opsonophagocytosis), degradation of the tissue barrier and spread within the human host (15).

Our patient presented with varicella and GAS associated NF and developed, as a further complication, IE which finally resulted in rupture of chordae tendineae of the mitral valve needing cardiac surgery. He did not have pre-existing heart disease. In general IE is a rare entity in children especially without pre-existing heart disease or central venous catheters *in situ* (16). The most frequently reported pathogens of bacterial IE in children are *S. aureus* (57%), *S. viridans* sp. (20%), and coagulase-negative staphylococci (14%) (10, 11). *S. pyogenes* is rarely (<3%) described as the cause of IE in patients under the age of 21 years (11). The percentage of endocarditis in patients >10 years associated with invasive GAS reported in the United States between 2005 and 2012 was 1.5% (17). In France during a similar time period the rate was 0.6% in adult patients (18).

We performed a literature review, as described above with the usual limitations, and found a total of only 18 children since 1942 with GAS associated IE—our patient excluded (Table 1). The median age of these children was 5 years. In only four cases M serotyping was performed, however different serotypes were responsible for IE (31, 32). We did not perform serotyping in our case. Ogura et al. focused their review on M serotypes including adult patients but did not find any particular serotype association for IE (32). We wanted to compare our case with other pediatric cases in the literature focussing on antecedent illness, complications and outcome. Five patients (28%) had varicella as antecedent illness. None of them developed necrotising fasciitis due to bacterial superinfection of the skin. Fourteen (78%) of these children had no pre-existing cardiac abnormalities and six (33%) had mitral valve involvement; Three (17%) were lethal. In a Japanese nationwide survey of rupture of the chordae tendineae of the mitral valve in infants, 95 cases were reported between 1995 and 2013. Interestingly, most cases were diagnosed as idiopathic. One infant had IE caused by *S. epidermidis*. None of these 95 infants with rupture of the chordae tendineae of the mitral valve where GAS associated (33).

**Table 1:** Summary of pediatric cases of IE due to GAS as reported since 1942.

**Case/reference**	**Year**	**Age/sex**	**Antecedent illness**	**Pre-existing cardiac abnormalities**	**Site of vegetation**	**Antibiotic therapy (duration)**	**Complications**	**Outcome**
1 (19)	1942	2 year, M	None	None	Mitral valve	none	Splenomegaly; Bronchopneumonia; Painful swollen joints	Death
2 (20)	1975	6 month, M	Bullous skin lesions and GAS bacteraemia	None	Aortic valve	Penicillin (28 d)	Aortic insufficiency; Sinus of Valsalva aneurysm; Left-ventricular failure	No progression of cardiac abnormalities at 2 year follow-up
3 (21)	1977	14 year, M	Tonsillitis	VSD closed surgically at age 6, aortic stenosis	Aortic	Not documented	Myocardial abscesses; Rupture of sinus of Valsalva into left ventricle	not documented
4 (22)	1984	16 year, M	Pharyngitis	None	Aortic valve	Cephalothin (3 d) Erythromycin (1 d) Penicillin and Gentamycin (14 d)	Respiratory failure; Renal emboli and renal failure; Petechiae	Renal function recovered; No cardiac abnormalities
5 (23)	1984	2.5 year, M	Pharyngitis	Mild pulmonary stenosis	Aortic, mitral, and tricuspid valves	Penicillin (10 d) Nafcillin and Gentamycin (14 d)	Aortic insufficiency; Mitral valve papillary muscle necrosis; Left ventricular failure; Petechiae	Death
6 (24)	1988	4 month, F	Varicella	None	Aortic valve	Cefuroxime (4 d) Cefaclor (3 d) Ampicillin and Gentamycin (4 d)	Aortic insufficiency; Paraaortic abscess	Death
7 (25)	1988	4.5 year, F	None	None	Aortic valve	Cefotaxime (3 d) Penicillin (NA)	Aortic insufficiency; Sinus of Valsalva aneurysm	Small residual ventricular septal defect, asymptomatic at 3 year follow-up
8 (26)	1992	3 year, F	None	None	Interatrial septum	Ceftriaxone (1 d) Penicillin (42 d)	Respiratory failure; Right cerebral infarction; Petechiae	Left-sides weakness at 6 month follow-up; No cardiac abnormalities
9 (27)	1998	14 year, M	None	None	Mitral valve	Penicillin (42 d)	Right cerebral infarction	Left-sides weakness; No cardiac abnormalities at 2 year follow-up
10 (28)	1999	5 month, M	Varicella	None	Mitral valve	Ceftriaxone and vancomycin (1 d) Ampicillin (2 d) Penicillin (42 d)	Respiratory failure; Seizures; purpura	No cardiac abnormalities at 2 year follow-up
11 (29)	2000	4 year, M	None	None	Mitral valve	Drug not documented (42 d)	Mitral regurgitation; Necrosis of 2 toes requiring amputation	Recovered
12 (29)	2000	3 year, M	Varicella	None	Aortic valve	Drug not documented (42 d)	Aortic root abscess; Aortic valve perforation; Focal seizures	Recovered with left hemiplegia
13 (10)	2000	3 year, M	Varicella	None	Aortic valve	Ceftriaxone (1 d) Clindamycin (duration NA) Penicillin (42 d)	Aortic insufficiency Left ventricular dilatation	Recovered
14 (30)	2004	11 year, M	Varicella	None	Aortic valve Mitral valve	Clarithromycin (6 d) Amikacin (duration NA) Cefotoxime (duration NA) Vancomycin (duration NA) Penicillin (42 d) Gentamycin (28 d)	Splenomegaly Abscess of aortic ring	Recovered
15 (31)	2014	6 year, F	Pharyngitis	None	Mitral valve	Ceftriaxone (1 d) Vancomycin (1 d) Meropenem (1 d) Clindamycin (4 d) Penicillin (42 d)	Septic emboli in Head CT	Recovered
16 (27)	2014	8 month, F	Pneumonia (possible)	None	Tricuspid valve	Vancomycin (1 d) Cefotaxime (1 d) Clindamycin (5 d) Penicillin (42 d)	Respiratory failure Multi organ dysfunction	Recovered
17 (32)	2015	17 month, M	Fever	Past history of spontaneous closure of VSD	Right-sided membranous septal aneurysm	Ampicillin (duration NA) Cefotaxime (duration NA) Ampicillin (>28 d)	None	Recovered
18 (32)	2016	14 year, F	Left foot cellulitis	None	Mitral valve	Ampicillin (duration NA) Sulbactam (duration NA) Clindamycin (duration NA) Ampicillin (duration NA) Cefotaxime (duration NA)	DIC Several infarctions (left temporal cortex, kidney, spleen)	Recovered

*GAS, Group A Streptococci; NA, not available; DIC, disseminated intravascular coagulation*.

Due to the multiple complications and severity of disease observed in our case, an underlying immunodeficiency may be postulated. Our child had no “red flags” from his family history or past medical history. Screening investigations for a possible defect in his cellular or humoral immune system were unremarkable. HIV screening was negative too. Extended investigations to review complement functions we did not perform. We postulate that the temporarily induced immunodeficiency state by VZV, the virulence factors expressed by GAS and the high bacterial load facilitated by the NF gave room to the course of the complications of our case.

## Conclusion

We have not found any previous reports in our literature search describing a similar case with multiple complications after VZV infection, in particular *S. pyogenes* associated IE with rupture of chordae tendineae of the mitral valve, in a previously healthy child without cardiac abnormalities but necrotizing fasciitis. None of the patients in the reports with varicella suffered from necrotizing fasciitis. In general GAS superinfection after VZV and GAS associated NF is common. However, IE as a complication of VZV infection is rare and *S. pyogenes* as the causative pathogen of IE is uncommon (10). Our case report demonstrates the enormous potential of VZV and GAS to cause life threatening disease particularly in young children. Both VZV and GAS can impact the host's immune defense at different levels. Our case also highlights that NF in childhood is a potentially lethal complication due to bacterial invasion of subcutaneous tissue with production of endo- and exotoxins causing ischemia and necrosis of the tissue, facilitating further bacteraemic spread and impaired penetration of antibiotics. A high index of suspicion and rapid surgical intervention is therefore of utmost importance. This is unfortunate given that varicella is a vaccine-preventable disease that could be controlled by a general childhood immunization programme (4, 34).

## Data Availability Statement

All datasets generated for this study are included in the article/supplementary material.

## Ethics Statement

Written informed consent was obtained from the minor(s)' legal guardian/next of kin for the publication of any potentially identifiable images or data included in this article.

## Author Contributions

PS substantially contributed to the conception and design of this article, reviewed the literature, summarized and analyzed the literature data, and drafted the initial manuscript. She critically reviewed the manuscript for important intellectual content and reviewed and revised the manuscript. UH substantially contributed to the conception and design of this article, critically reviewed the manuscript for important intellectual content, and reviewed and revised the manuscript. MB substantially contributed to the conception and design of this article, supported the literature review, critically reviewed the manuscript for important intellectual content, and reviewed and revised the manuscript. All authors approved the final manuscript as submitted and agree to be accountable for all aspects of the work.

### Conflict of Interest

The authors declare that the research was conducted in the absence of any commercial or financial relationships that could be construed as a potential conflict of interest.
